# Supplementary material for: HIVprotI: an integrated web based platform for prediction and design of HIV proteins inhibitors
Source: J Cheminform. 2018 Mar 9;10:12. doi: 10.1186/s13321-018-0266-y (PMC5845081; doi:10.1186/s13321-018-0266-y)
Supplement: Supplementary file 2 — Additional file 2. Source code of HIVProtI web server. [file 13321_2018_266_MOESM2_ESM.zip › HIVprotI_Source-code/hivprotI/weka-3-6-6/doc/weka/associations/CARuleMiner.html]

CARuleMiner


---


|  |  |  |
| --- | --- | --- |
| **Overview** \| **Package** \| **Class** \| **Tree** \| **Deprecated** \| **Index** \| **Help** \| **Weka's home** | |  |
| **PREV CLASS**   **NEXT CLASS** | **FRAMES**    **NO FRAMES**     **All Classes** |  |
| SUMMARY: NESTED \| FIELD \| CONSTR \| METHOD | DETAIL: FIELD \| CONSTR \| METHOD |  |


---


## weka.associations Interface CARuleMiner

**All Superinterfaces:**: OptionHandler

**All Known Implementing Classes:**: Apriori, PredictiveApriori

---

``` public interface CARuleMiner extends OptionHandler ```

Interface for learning class association rules. All schemes for learning
class association rules implemement this interface.

**Version:**
:   $Revision: 1.3 $

**Author:**
:   Stefan Mutter (mutter@cs.waikato.ac.nz)

---

| **Method Summary** | |
| --- | --- |
| `Instances` | `getInstancesNoClass()`             Gets the instances without the class attribute |
| `Instances` | `getInstancesOnlyClass()`             Gets the class attribute and its values for all instances |
| `java.lang.String` | `metricString()`             Gets name of the scoring metric used for car mining |
| `FastVector[]` | `mineCARs(Instances data)`             Method for mining class association rules. |
| `void` | `setClassIndex(int index)`             Sets the class index for the class association rule miner |

**Methods inherited from interface weka.core.OptionHandler**

`getOptions, listOptions, setOptions`

**Method Detail**

### mineCARs

```
FastVector[] mineCARs(Instances data)
                      throws java.lang.Exception
```

:   Method for mining class association rules.
    Must initialize all fields of the CARuleMiner that are not being set via options (ie. multiple calls of mineCARs
    must always lead to the same result). Must not change the dataset in any way.

    :   **Parameters:**: `data` - the insatnces for which class association rules are mined **Returns:**: class association rules and their scoring metric in an FastVector array **Throws:**: `java.lang.Exception` - throws exception if class association rules cannot be mined

---


### getInstancesNoClass

```
Instances getInstancesNoClass()
```

:   Gets the instances without the class attribute

    :   **Returns:**: the instances withoput the class attribute

---


### getInstancesOnlyClass

```
Instances getInstancesOnlyClass()
```

:   Gets the class attribute and its values for all instances

    :   **Returns:**: the class attribute and its values for all instances

---


### metricString

```
java.lang.String metricString()
```

:   Gets name of the scoring metric used for car mining

    :   **Returns:**: string containing the name of the scoring metric

---


### setClassIndex

```
void setClassIndex(int index)
```

:   Sets the class index for the class association rule miner

    :   **Parameters:**: `index` - the class index


---


|  |  |  |
| --- | --- | --- |
| **Overview** \| **Package** \| **Class** \| **Tree** \| **Deprecated** \| **Index** \| **Help** \| **Weka's home** | |  |
| **PREV CLASS**   **NEXT CLASS** | **FRAMES**    **NO FRAMES**     **All Classes** |  |
| SUMMARY: NESTED \| FIELD \| CONSTR \| METHOD | DETAIL: FIELD \| CONSTR \| METHOD |  |


---
